# Cultural representations of dementia

**DOI:** 10.1371/journal.pmed.1002274

**Published:** 2017-03-28

**Authors:** Alexandra Hillman, Joanna Latimer

**Affiliations:** 1 School of Social Sciences, Cardiff University, Cardiff, United Kingdom; 2 Science & Technology Studies Unit, Department of Sociology, University of York, Heslington, York, United Kingdom

## Abstract

In a Perspective, Alexandra Hillman and Joanna Latimer discuss cultural representations of dementia in the media, film, and literature.

Dementia has been positioned as one of the global health priorities of our age [1]. This positioning has been accompanied by an increased attention from governments, biological and clinical sciences, practitioners, care providers, and the wider public, laying the foundations for a cultural preoccupation with loss of memory. As Margaret Lock [2], an American cultural anthropologist, suggests, Alzheimer disease (AD) and other Alzheimer-like dementias personify all that is most feared about growing old—a fear widely expressed in research on aging and experiences of aging (e.g., [3]).

Dementia itself is, in part, a culturally determined phenomena, one that relies upon biomedicine’s ability to name and give form to a collection of changes, behaviours, and experiences. Anthropologist Lawrence Cohen’s [4] research in India, for example, describes a community in which dementia was previously not culturally marked as something associated with suffering or warranting medical intervention. Following the growth of clinical professionals like geriatricians, alongside global media and marketization of lifestyle interventions directed at older people, local perceptions of “the elderly” changed and created a socially constructed, yet locally experienced, dementia “epidemic” [5]. Indeed, cross-cultural research identifies how understandings of dementia as either a natural part of the aging process or as a result of brain disease are culturally shaped [6,7]. Lock’s [2] work, although not a cross-cultural comparison, is careful to consider the sociohistorical and cultural conditions—as well as the scientific developments—that have informed the separation of AD from the “normal” aging process. Similar to the growth in diagnosable mental illnesses that occurred at the turn of the twentieth century [8], dementia represents an increasingly wide compendium descriptor for many different effects [9]. Differences in the ways in which dementia is conceptualised may also be shaped by social location. Although work on social class and dementia tends to be restricted to differences in associated risk factors for developing the condition or different levels of access to diagnosis, care, and support, there is a growing recognition that social class identities have a significant role to play in the ways in which dementia is both experienced and conceptualised [10].

This body of research not only highlights the diverse ways in which dementia is experienced but also suggests that the cultural meanings attached to dementia—even within societies—are also not universal [11]. Meanings of dementia are interpreted, embodied, or resisted by people in their social contexts, and these processes are shaped according to their social location (gender, social class, and ethnicity) and their individual biography [12–14]. As Cohen’s research in India identified, cultural representations of dementia also become entwined with other social meanings, such as the social expectations of aging [15]. Recognising this complexity goes some way to helping social scientists understand how particular meanings associated with dementia gain traction over others, what the implications of these cultural representations are for those living with the condition, and how these implications may differ according to a person’s social location.

It is in this tradition that researchers are interested in studying the proliferation of stories that seek to represent what dementia is, what it is like to have it, or what it is like to live with those who do. In Euro-American cultures at least, this proliferation of dementia stories has been reflected in mainstream press and television news as well as in works of fiction across TV, film, and literature (e.g., [16]). Accounts of dementia, particularly from politicians and in the press, tend to evoke frightening images, presenting it as a kind of living death for its sufferers—the body remains but the person is lost [17]. Such images include associating people with AD as zombies, creating a reaction of revulsion and fear. The language of loss and determinism pervade these dominant cultural representations, describing the growing incidents of dementia amongst populations as a rising tide, suggesting an unstoppable force. Smaller, personal stories that attempt to shed light on the experience of dementia tend to focus on the extremes of the disease, making dementia freakish, something that happens to “them” rather than “us” [18, 19].

Running in parallel to these stories are representations of hope—not for the possibility of living well with the condition, but instead to eradicate it. These stories tend to focus on a potential miracle cure that might prevent cognitive decline as we age. Peel [13] describes this dichotomy in United Kingdom mainstream press as a “panic blame discourse” that reproduces images of dementia as inevitable loss and decline while simultaneously telling stories about strategies for staving off the disease. This reframes the condition as something amenable to individual behaviours and choices and thus a failing of those afflicted to age successfully—a judgement that may unevenly befall on the most economically and physically vulnerable.

Alongside the press attention, we see TV, film, and literature increasingly feeding into cultural understandings of dementia (for example, *Emmerdale Farm* ITV 2016 [20]; *Wallander* BBC 2016 [21]; *Elizabeth is Missing* by Emma Healy, 2014 [22]). One film, *Iris*, by Richard Eyre based on the book *Iris*, *A Memoir and Elegy for Iris Murdoch*, 1998 [23], written by John Bayley, offers a nuanced message (see also [24]). Rather than depicting dementia only in terms of a diseased brain, and personhood as only associated with this rather impoverished understanding, dementia is also portrayed as disordering identities and ways of relating to others that transform, rather than just obliterate, the person. In particular, the film shows how dementia challenges the complex social processes that produce, the appearance at least, of the discrete individual, including cooperation, or what sociologists such as Erving Goffman observe as a person’s capacity to fit in, get along, and pass as a member. Thus, the film helps to trouble and challenge a discourse of waging a war on dementia. Dementia in art has also been used as a vehicle to explore some fundamental existential questions about what forms the basis of our being in the world. The film *Robot and Frank*, 2013 [25], set in the near future, depicts a relationship between Frank, who has dementia, and his robot procured by Frank’s children to provide living-in-home help for Frank as his dementia progresses. Through this relationship, the film raises interesting questions about the connections between memory, identity, and humanity: what is it that makes us human? In particular, Frank’s being in the world is shown to be somewhat prereflective [26]: it is embodied, emotional, and, finally, social. Iris, Frank, and, more recently, Alice from the novel and film *Still Alice*, 2015 [27], embody the contradictions that make up contemporary images of dementia. On the one hand, they reflect our fears of obliteration, the tragedy of a gradual chipping away of our humanity; while on the other hand, we are met with characters who remain present as a moral force, who feel pleasure and pain, who have emotional responses and connections to their social and material worlds. Perhaps the effects of the disease are less of a tragedy than the torment of our own contradictions. For example, at the end of the film *Iris*, we see her husband and her friends accepting that she is someone different, with Iris herself happy in a new environment where she is able to be the person she has become rather than who she once was.

There are many ways of unlocking and challenging the assumptions that underpin cultural understandings of dementia, as many of these depictions in film and literature are beginning to do. There are also worldwide programs pressing ways to destigmatise dementia (e.g., [28]), with new social formations creating possibilities for living with dementia differently, such as dementia-friendly communities across Europe, Japan, and the United States. In these ways, alongside biomedical and public health programs for addressing whether there are disease processes that underpin dementia and which can be remedied, different communities are finding alternative ways of being in the world that are more accepting and embracing of the kinds of disruptions that dementia can produce.
